# The novel pseudo-compound transposon Tn7086 carries aminoglycoside resistance genes in Enterococcus faecalis

**DOI:** 10.1099/mgen.0.001680

**Published:** 2026-04-07

**Authors:** Lorenzo Colombini, Mariana Tirziu, Stefano De Giorgi, Anna Maria Cuppone, Giuseppe Sangiorgio, Carmelo Bonomo, Dafne Bongiorno, Stefania Stefani, Susanna Ricci, Gianni Pozzi, Francesco Santoro, Francesco Iannelli

**Affiliations:** 1 Laboratory of Molecular Microbiology and Biotechnology, Department of Medical Biotechnologies, University of Siena, 53100 Siena, Italy; 2 Medical Molecular Microbiology and Antibiotic Resistance Laboratory (MMARLab), Department of Biomedical and Biotechnological Sciences (BIOMETEC), University of Catania, 95123 Catania, Italy; 3 Mycobacteriology and Cell Response to Infection, Siena University Hospital, Siena, Italy

**Keywords:** *Enterococcus faecalis*, pseudo-compound transposon, mobilome, antimicrobial resistance genes, complete genome

## Abstract

*Enterococcus faecalis* is a member of the human gut microbiota and a pathogen responsible for mild and severe infections. Here, genome analysis of *E. faecalis* strains isolated from different body sites revealed the presence of a novel family of IS*1216E*-flanked pseudo-compound transposons carrying aminoglycoside resistance genes and other resistance determinants. The representative element, named Tn*7086*, is 25,380 bp long, contains 27 ORFs and shows a mosaic structure containing (i) the macrolide–lincosamide–streptogramin resistance gene *erm*(B), (ii) the aminoglycoside–streptothricin resistance gene cluster *ant(6′)-Ia–sat4–aph(3′)-IIIa*, (iii) the gentamicin resistance determinant *acc(6′)-aph(2*″) and (iv) a toxin–antitoxin cassette. Tn*7086* family members contain deletions and/or insertions including three DNA segments, two of which carry antimicrobial resistance genes. All elements integrate downstream of a conserved 8-bp target site within the chromosomal *panE* gene, located between *lysR* and *rbgA* and encoding a 2-dehydropantoate 2-reductase. Genome-wide analysis of 646 complete *E. faecalis* genomes showed *panE* disruption in 12.7% of isolates due to the presence of Tn*7086* family members (10.7%) or *IS1216E* (2%), while an intact *panE* gene was found in the other genomes. Element integration produced either target-site duplication or DNA deletions, with or without the target site. PCR and sequencing analysis showed that Tn*7086* and Tn*7086*-like elements excise from the chromosome and produce circular translocatable units at frequencies of 1.24±0.03 to 22.4±17.7 copies per 10^6^ chromosomes. In conclusion, we describe the novel Tn*7086* family of IS*1216E*-flanked pseudo-compound transposons in *E. faecalis*, which carry multiple antimicrobial resistance genes, integrate at a specific chromosomal site within *panE* and are capable of excision to form circular translocatable units.

**Impact Statement**

In this work, through comparative genomics, we identified Tn*7086* as the progenitor of a novel family of pseudo-compound transposons that reshape the *Enterococcus faecalis* genome. Tn*7086* family members have a mosaic structure, can accumulate multiple antimicrobial resistance determinants, are flanked by IS*1216E* direct repeats and integrate at the *panE* conserved chromosomal locus. Genome-wide analyses of public databases revealed the presence of different Tn*7086* family members in 10.7% of *E. faecalis* genomes and further variability at the *panE* locus in 2% of the genomes, associated with the presence of IS*1216E*. Finally, we demonstrated that Tn*7086* can form circular translocatable units containing one copy of IS*1216E*, likely playing a key role in both intra- and intercellular transposition of the element, in the generation of novel mosaic structures and in the dissemination of antibiotic resistance in this important pathogen.

## Data Summary

The complete genome sequences and the Nanopore and Illumina sequencing reads are available under National Center for Biotechnology Information BioProject accession no. PRJNA1209846. Complete genomes of strains 2819, 4638, 4774, 5034, 5245 and 5410 are available under GenBank accession nos. CP181214–CP181242, while the Tn*7086* sequence is available under GenBank accession no. PV941854.

## Introduction

*Enterococcus faecalis* is a member of the human gut microbiota and also a pathogen responsible for both mild and severe infections, such as urinary tract infections, peritonitis, endocarditis and sepsis [1,2]. *E. faecalis* infections are generally treated with *β*-lactams or glycopeptides; however, severe cases often require a combination of *β*-lactams or glycopeptides with aminoglycosides, typically gentamicin [3,6]. A low level of resistance to aminoglycosides is an intrinsic characteristic of enterococci, consequent to their metabolism that limits drug uptake [7]. Inhibition of cell wall synthesis due to *β*-lactams or glycopeptide action increases aminoglycoside uptake, resulting in synergistic killing of enterococci [8]. Acquisition of aminoglycoside-modifying enzyme (AME) genes results in high levels of resistance to aminoglycosides, limiting the antibiotic synergistic effect [5]. AME genes include the *aac(6′)-aph(2*″) gene, which encodes the bifunctional enzyme AAC(6′)-APH(2″) conferring resistance to the majority of aminoglycosides routinely used in clinical practice [8]. Both the *ant(6′)-Ia* encoding ANT(6′)-Ia aminoglycoside nucleotidyltransferase and *aph(3′)-IIIa* encoding 3′-O-phosphotransferase are widespread AME genes responsible for resistance to streptomycin and kanamycin, respectively [9]. Mobile genetic elements (MGEs), collectively referred to as the mobilome, contribute to the spread of antimicrobial resistance and virulence genes [10]. *E. faecalis* mobilome includes integrative and conjugative elements, such as the Tn*916*/Tn*1545* family of conjugative transposons, the Tn*3/21* family of non-conjugative transposons and composite transposons [11,13]. Furthermore, it includes pseudo-compound transposons, which are DNA sequences flanked by directly oriented insertion sequence (IS) and capable of intracellular transposition to new genomic sites by a two-step process involving a circular translocatable unit [14]. The assembly of pseudo-compound transposons is mainly mediated by IS*6*/IS*26*-family elements, such as IS*26* in Gram-negative bacteria and IS*257*/IS*431* or IS*1216* in Gram-positive bacteria [15]. Our previous study performed on 41 clinical isolates of *E. faecalis* identified high-level aminoglycoside resistance in 8 strains, with AME genes located on either chromosomes (6/8 strains) or plasmids (2/8 strains) [16]. In this study, genome sequence analysis of the six *E. faecalis* strains carrying chromosomal AME genes and three additional gentamicin-resistant blood isolates revealed the presence of the novel Tn*7086* family of pseudo-compound transposons carrying AME genes along with other antimicrobial resistance determinants and sharing the same specific chromosomal integration site.

## Methods

### Bacterial strains, growth conditions and minimal inhibitory concentration determination

*E. faecalis* strains were isolated from genital samples of infertile patients or from patients with sepsis (Table S1, available in the online Supplementary Material 1) [16,17]. *E. faecalis* strain OG1RF was used as a reference control strain [18]. Bacteria were grown in brain heart infusion (BHI) (Oxoid, Milan, Italy) medium or in BHI supplemented with 1.5% agar (BD Difco) and 3% defibrinated sheep blood (Liofilchem) in the presence of 5% CO_2_ at 37 °C. Antibiotic susceptibility was determined by broth microdilution minimal inhibitory concentration (MIC) assay as already described [19,20]. Briefly, bacteria were grown in BHI medium until the exponential phase (OD_590_=0.3, corresponding to ~10^8^ c.f.u. ml^−1^). Culture aliquots were diluted 1:100 in BHI (10^6^ c.f.u. ml^−1^), and 100 µl was added to a 96-well microplate containing 100 µl of twofold serial dilutions of antibiotic to a final concentration of 5×10^5^ c.f.u. ml^−1^ in each well. Plates were incubated at 37 °C and visually analysed after 18 h. *E. faecalis* ATCC 29212 was used as a quality control strain.

### Genomic DNA purification and sequencing

Bacteria were grown to late exponential phase (OD_590_ of ~2.0), and high-molecular-weight genomic DNA was purified using a raffinose-based method [21,23]. DNA was quantified using a Qubit 4.0 Fluorometer (Invitrogen, Life Technologies, Carlsbad, CA, USA) with the Qubit dsDNA BR Assay Kit (Thermo Fisher Scientific) and a spectrophotometer (Implen, Munich, Germany), while DNA integrity and size were assessed by agarose gel electrophoresis (0.6% Seakem LE, Lonza, Rockland, ME, USA) with 0.5× TBE running buffer. Whole-genome sequencing was performed using both Illumina and Nanopore sequencing technologies as reported [24,25]. Illumina sequencing was carried out at MicrobesNG (University of Birmingham, UK) using the Nextera XT library preparation kit (Illumina Inc.) on a NovaSeq 6000 device (Illumina, 2×250 bp paired-end sequencing). For Nanopore sequencing, genomic DNA was size-selected with 0.5 volumes of AMPure XP beads (Beckman Coulter, Milano, Italy) and used as input for library preparation with the SQK-LSK 108 kit (Oxford Nanopore Technologies). Sequencing was performed on a GridION X5 device (Oxford Nanopore Technologies) using an R9.4 flow cell (FLO-MIN106) (Oxford Nanopore Technologies). Real-time base calling was performed with Guppy v3.2.6 (Oxford Nanopore Technologies), filtering out reads with a Q7 quality cut-off.

### Genome assembly and analysis

Complete genome assembly was carried out as described [25,26]. Briefly, Nanopore reads were filtered to obtain 30× coverage, considering 2.7 Mb as the genome size estimate using Filtlong v0.2.1 software (https://github.com/rrwick/Filtlong) with parameter ‘*--target_bases*’ and then assembled using Flye v2.9.3-b1797 (https://github.com/mikolmogorov/Flye). The resulting circular contigs were polished with Medaka v1.11.3 (https://github.com/Nanoporetech/medaka) using the filtered Nanopore reads, followed by two polishing rounds with Pilon v1.24 (https://github.com/broadinstitute/pilon) using Illumina reads trimmed using Trimmomatic v0.30 (https://github.com/usadellab/Trimmomatic). Assembly completeness was assessed with Bandage v.0.8.1 (https://github.com/rrwick/Bandage), whereas assembly quality was evaluated using Ideel (https://github.com/mw55309/ideel) and CheckM v1.1.3 (https://github.com/Ecogenomics/CheckM). Genomes were automatically annotated with the National Center for Biotechnology Information (NCBI) Prokaryotic Genome Annotation Pipeline v6.9 [27]. The genome sequence of *E. faecalis* strain OG1RF (GenBank accession number CP002621) was used as a reference for genome comparison. DNA sequence analysis was performed with Artemis/ACT v17.0.1 (http://sanger-pathogens.github.io/Artemis/). Antibiotic resistance gene analysis was performed using RGI (Resistance Gene Identifier) (v6.0.3) (https://card.mcmaster.ca/analyze/rgi), based on CARD v3.3.0 (Comprehensive Antibiotic Resistance Database), with the parameter ‘*-loose_criteria=no*’. Manual annotation of MGEs was carried out by blast homology search of the databases available at the NCBI (https://blast.ncbi.nlm.nih.gov/Blast.cgi?PAGE=Proteins) and the Pfam protein family database (available under the InterPro consortium, https://www.ebi.ac.uk/interpro/search/sequence/). Transposon names were assigned by curators of the Tn Registry website (https://transposon.lstmed.ac.uk/tn-registry). Default parameters were used for all software unless otherwise specified.

### PCR and amplicon sequencing

PCR and direct PCR sequencing were performed as previously described [18,28, 29]. Oligonucleotide primers and their positions are listed in Table S2 (Supplementary Material 1). Transposon circular forms were detected using divergent primers directed at the ends of the transposons, while reconstitution of target sites was investigated using primers directed at the chromosomal junction fragments, as reported [30,31]. Briefly, quantitative PCR (qPCR) was performed using the KAPA SYBR FAST qPCR kit Master Mix Universal (2X) (Merck) on a LightCycler 1.5 apparatus (Roche Diagnostics). The real-time PCR mixture, in a final volume of 20 µl, contained 1×KAPA SYBR FAST qPCR reaction mix, 5 pmol of each primer and 20 ng of purified high-molecular-weight bacterial DNA. The thermal profile included an initial 4 min denaturation step at 95° C, followed by 40 cycles of denaturation (10 s at 95 °C), annealing (15 s at 61 °C) and elongation (3 min at 72 °C), with elongation extended to 4 min for strains 4774, 5034 and 5410. The temperature transition rate was 20 °C/s for denaturation and annealing and 5 °C/s for elongation. Primer pair IF1344/IF1345 generating amplicons of 1.7 or 2.3 kbp was used for reconstituted integration site quantification in each strain, while to quantify transposon circular transposable units, primer pairs IF1396/IF1418 (3176 bp amplicon), IF1404/IF1405 (3506 bp), IF1406/IF1418 (2850 bp), IF1482/IF1400 (3413 bp), IF1484/IF1418 (2542 bp), IF1400/IF1418 (3125 bp), IF1404/IF1405 (4244 bp) and IF1418/IF1400 (3132 bp) were used in strains 2819, 4638, 5245, 5410, 5034, 4774, Ef-1549 and Ef-2580, respectively. A 1,406 bp fragment of the *gyrB* gene, obtained with primers IF943/IF1495, was used to standardize results. The standard curve was generated by plotting the threshold cycle against chromosomal copy number using serial dilutions of *E. faecalis* OG1RF DNA at known concentrations. Melting curves were analysed to distinguish amplified products from primer dimers.

## Results

### Identification of the Tn*7086* family of pseudo-compound transposons carrying aminoglycoside resistance genes in *E. faecalis* clinical isolates

Our previous study reported that six *E. faecalis* clinical isolates (strains 2819, 4638, 4774, 5034, 5245 and 5410), presented with high-level resistance to aminoglycosides (MIC of gentamicin ranged from 1,024 to 8,192 µg ml^−1^), were genetically related and carried the *aac(6′)-aph(2*″) aminoglycoside-modifying enzyme gene in the chromosome [16] (Table S1). Two *E. faecalis* blood isolates, Ef-1549 and Ef-2580, also carried the *aac(6′)-aph(2*″) AME gene and showed the same pattern of antimicrobial resistance (MIC ≥4,096 µg ml^−1^ for gentamicin). Whole-genome sequence of these eight strains and of the aminoglycoside-susceptible *E. faecalis* blood isolate Ef-871 was obtained by hybrid assembly of Illumina and Nanopore data. Genome comparison analysis of the eight resistant clinical isolates and the reference laboratory strain OG1RF [32] revealed the presence of a novel family of pseudo-compound transposons carrying the aminoglycoside resistance determinant *aac(6′)-aph(2*″) and other resistance genes. The elements were flanked by two copies of IS*1216E* and were always found integrated at the same chromosomal *panE* gene coding for a 2-dehydropantoate 2-reductase, located between *lysR* and *rbgA*. Based on comparative sequence analysis, the element of isolate Ef-1549, which we named Tn*7086*, was considered the progenitor of this transposon family, since transposon variants found in other isolates likely arose through insertions and deletions of different DNA segments.

### The pseudo-compound transposon Tn*7086*

**Fig. 1.**
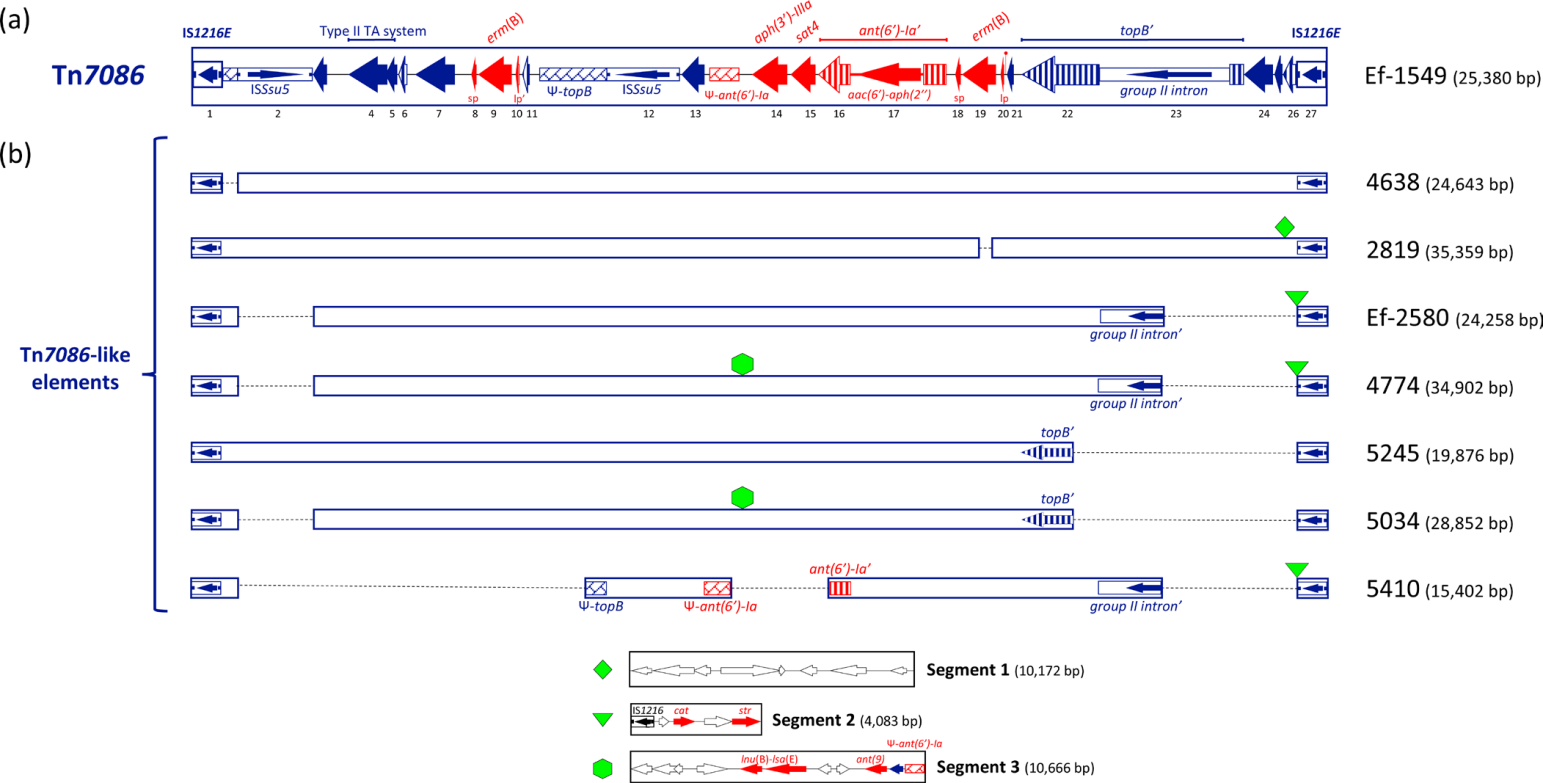
(**a**) Schematic structure of *E. faecalis* Tn*7086* and (**b**) comparison with Tn*7086*-like elements. (**a**) Tn*7086* is a 25,380-bp-long pseudo-compound transposon with an average GC content of 34.69% and containing 27 ORFs including (**i**) the macrolide–lincosamide–streptogramin resistance gene *erm*(B), (ii) the aminoglycoside–streptothricin resistance *ant(6′)-Ia–sat4–aph(3′)-IIIa* gene cluster, (iii) the gentamicin resistance determinant *acc(6′)-aph(2*″) and (iv) a toxin–antitoxin (TA) gene cassette. (**b**) Tn*7086* is compared with 7 *E. faecalis* Tn*7086*-like elements. The names of isolates carrying the element are reported on the right of the figure. For a better alignment of the sequences, the elements were devoid of insertions indicated by solid geometric shapes, while deletions were indicated by dashed lines. The elements range in size from 15,402 bp to 35,359 bp and are delimited by two copies of IS*1216E* arranged as direct repeats. Insertions include (**i**) the 10,172-bp pseudo-compound transposon Tn*7087* with an average GC content of 31.69% and containing eight ORFs and (ii) segment 1 and segment 2 carrying antimicrobial resistance genes. ORFs and their direction of transcription are represented by arrows, and annotated ORFs are indicated only by their numbers. Antimicrobial resistance genes are represented by red arrows. ISs and group II introns are reported as boxed black arrows. Disrupted genes are reported as striped arrows, while pseudogenes are reported as pattern-filled boxes.

Tn*7086* is 25,380 bp in length with an overall GC content (34.69%) lower than the genome average of 37.75%, spanning nts 1,527,931 to 1,553,310 in the *E. faecalis* Ef-1549 chromosome. Tn*7086* contains 27 ORFs, of which 26 are transcribed in the same direction, and *orf15* and *orf20* start with a GTG codon (Fig. 1a). Manual homology-based annotation attributed a putative function to 25 out of the 27 ORFs (Table 1). Tn*7086* is flanked by two direct repeats of the insertion sequence IS*1216E* of the IS*6* family (https://isfinder.biotoul.fr/scripts/ficheIS.php?ident=99). Each IS*1216E* is 808 bp long and contains a 681-bp transposase gene (*orf1* and *orf27*). In addition to IS*1216E*, Tn*7086* contains a group II intron (*orf23*) [33] and two identical IS*Ssu5* elements of the IS*1380* family (https://isfinder.biotoul.fr/scripts/ficheIS.php?name=ISSsu5), arranged in opposite directions and containing a 1,320-bp-long transposase gene (*orf2* and *orf12*). Tn*7086* is characterized by a mosaic structure containing several antimicrobial resistance genes, previously identified in other characterized transposons, including (i) the macrolide–lincosamide–streptogramin resistance gene *erm*(B) of *E. faecalis* Tn*917* [34], present in two copies (*orf9* and *orf19*); (ii) the aminoglycoside–streptothricin resistance gene cluster *ant(6′)-Ia–sat4–aph(3′)-IIIa* (*orf16-orf15-orf14*) of *Staphylococcus aureus* Tn*5405* [35]; and (iii) the gentamicin resistance determinant *acc(6′)-aph(2*″) (*orf17*) of *S. aureus* Tn*4001* [36]. Two ORFs, namely *orf16* (*ant(6′)-Ia*) and *orf22* (*topB*), are disrupted by the insertion of a 1,918-bp fragment containing the 1,440-bp *acc(6′)-aph(2*″) coding sequence and by the 2,766-bp group II intron, respectively. Pseudogenes, Ψ-*topB* and Ψ-*ant(6′)-Ia*, are homologous to the 3′ end of the DNA topoisomerase gene *topB* and to the 3′ end of the aminoglycoside 6-adenylyltransferase *ant(6′)-Ia*, respectively. Tn*7086* also contains genes typically involved in plasmid maintenance, including *orf4* and *orf5*, constituting a toxin–antitoxin system [37,38] and *orf7* encoding a plasmid partition protein [39].

**Table 1.** Annotated ORFs of the Tn*7086* pseudo-compound transposon

ORF (aa)*	Predicted protein	Homologous protein ID/origin identity (%) (*E* value)†	Pfam domain (aa)‡ (*E* value)
*orf1* (226)	IS*1216E*, transposase, IS*6* family	AAC44739.1/*E.faecium* 223/225 (99%) (3e−171)	
*orf2* (439)	IS*Ssu5*, transposase, IS*1380* family	ABP92124.1/*S.suis* 439/439 (100%) (0.0)	
*orf4* (287)	Zeta toxin	1GVN_B/*S. pyogenes* 278/287 (97%) (0.0)	Zeta_toxin (27–214) (3.8e−42)
*orf5* (90)	Epsilon antitoxin	1GVN_A/*S. pyogenes* 90/90 (100%) (3e−59)	Epsilon_antitox (4–83) (5.6e−26)
*orf6* (60)	Omega transcriptional repressor, 5′ partial		Omega_Repress (3–60) (1.6e−36)
*orf7* (298)	Plasmid partition protein A, Soj homologue	2OZE_A/*S. suis* 293/298 (98%) (0.0)	AAA_31 (37–213) (7.9e−26)
*orf8* (43)	23S rRNA adenine N-6-methyltransferase, signal peptide	AAA27453.1/*E. faecalis* Tn*917* 43/43 (100%) (9e−27)	
*orf9* (245)	23S rRNA adenine N-6-methyltransferase	AAA27452.2/*E. faecalis* Tn*917* 243/245 (99%) (0)	RrnaAD (2–240) (5.5e−79)
*orf10* (23)	23S rRNA methyltransferase leader peptide, 3′ partial	AAA27451.2/*E. faecalis* Tn*917* 26/31 (84%) (2e−12)	ErmC (1–23) (2.0e−14)
*orf11* (41)	Omega transcriptional repressor, 5′ partial		Omega_Repress (3–41) (2.1e−21)
*orf12* (439)	IS*Ssu5*, transposase, IS*1380* family	ABP92124.1/*S.suis* 439/439 (100%) (0.0)	
*orf13* (175)	Adenine phosphoribosyltransferase		Pribosyltran (36–151) (4.7e−11)
*orf14* (264)	Aminoglycoside 3′-phosphotransferase	AF330699.1/*E. faecium* 263/264 (99%) (0.0)	APH (26–256) (3.0e−26)
*orf15* (180)	Streptogramin A acetyltransferase	AF330699.1/*E. faecium* 180/180 (100%) (1e−122)	Acetyltransf_1 (42–153) (6.8e−21)
*orf16*′ (267)	Aminoglycoside 6- adenylyltransferase, 3′ partial	AF330699.1/*E. faecium* 233/233 (100%) (3e−172)	Adenyl_transf (3–242) (8.8e−98)
*orf17* (479)	Bifunctional acetyltransferase-phosphotransferase	GU565967.1/*S. aureus* pSK1 479/479 (100%) (0.0)	APH (205–440) (6.3e−27) Acetyltransf_8 (15–156) (7.8e−16)
*orf16′* (37)	Aminoglycoside 6- adenylyltransferase, 5′ partial	AF330699.1/*E. faecium* 37/37 (100%) (2e−20)	Adenyl_transf (1–37) (2.2e−9)
*orf18* (43)	23S rRNA adenine N-6-methyltransferase, signal peptide	AAA27453.1/*E. faecalis* Tn*917* 42/43 (98%) (3e−30)	
*orf19* (245)	23S rRNA adenine N-6-methyltransferase	AAA27452.2/*E. faecalis* Tn*917* 243/245 (99%) (0)	RrnaAD (2–240) (5.6E−9)
*orf20* (28)	23S rRNA adenine N-6-methyltransferase, leader peptide		
*orf21* (79)	Omega transcriptional repressor		Omega_Repress (3–61) (1.2e−33)
*orf22*′ (600)	DNA topoisomerase type IA, 3′ partial	EGQ1712264.1/*S. pseudointermedius* 557/557 (100%) (0.0)	Topoisom_bac (516–545) (1.4e−7)
*orf23* (628)	Group II intron reverse transcriptase/maturase	WP_160459188.1*/S*.*aureus* 626/628 (99%) (0.0)	RVT_1 (105–352) (6.3e−32)
*orf22′* (115)	DNA topoisomerase type IA (DNA primase domain), 5′ partial	EGQ1712264.1/*S. pseudointermedius* 116/117 (99%) (9e−82)	Toprim (3–115) (1.4e−9)
*orf24* (205)	DNA resolvase (recombinase)		Resolvase (4–141) (1.5e−28)
*orf26* (88)	Transcriptional regulator, 3′ partial		Tscrpt_reg_MerR_DNA-bd (20–80) (0.0021)
*orf27* (226)	IS*1216E*, transposase, IS*6* family	AAC44739.1/*E.faecium* 223/225 (99%) (3e−171)	

*The number of amino acids of the predicted protein is shown in parentheses.

†Determined by compositional matrix adjustment.

‡Numbers in parentheses indicate the region of the protein homologous to the Pfam domain.

### Comparative analysis of the Tn*7086* family transposons

In addition to the Tn*7086* found in the Ef-1549 isolate, a Tn*7086*-like element was found in each aminoglycoside-resistant isolate, with sizes varying between 15,402 bp and 35,359 bp (Fig. 1b). Comparison of Tn*7086* with pseudo-compound transposons found in the other strains revealed the insertion of three distinct DNA fragments in five elements. A 10,172-bp-long DNA insert, named Segment 1, was found integrated downstream of *orf25* within the Tn*7086*-like element of isolate 2819. A 4,083-bp-long DNA insert, named Segment 2, was found in the Tn*7086*-like element of isolates Ef-2580, 4774 and 5410. Segment 2 was located at nt 24,566 of Tn*7086*, contained an 808-bp IS*1216E* and carried the *cat* and *str* genes, for chloramphenicol and streptomycin resistance (Fig. 1b and Table 2). Segment 3, a 10,666-bp DNA fragment carrying the lincosamide resistance genes *lnu*(B)*-lsa*(E) and the spectinomycin resistance gene *ant* [9], was found at nt 10,828 of isolates 4774 and 5034. Insertion of Segment 3 produced a 1,153-bp direct duplication involving *orf13* and Ψ-*ant(6′)-Ia* (Fig. 1b and Table 2). Sequence comparison also revealed deletions of different DNA regions within the Tn*7086*-like element sequences. Deletions at the 5′ end involved (i) the IS*Ssu5* element (1,687 bp) in isolates 4774 and 5034, (ii) an 8,072-bp DNA region spanning *orf2* to *orf11* (nts 809 to 8,831) in isolate 5410 and (iii) 244 bp of the *erm*(B) gene (*orf19*) in isolate 2819. At the 3′ end, the following DNA deletions were detected: (i) a 3,188-bp fragment spanning *orf23* to *orf26* (nts 20,994 to 24,565) in isolates Ef-2580, 4774, and 5410 and (ii) a 5,163-bp DNA region encompassing *orf22* to *orf26* (nts 19,019 to 24,565) in isolates 5245 and 5034. Finally, the Tn*7086*-like element of isolate 5410 contained a 1,973-bp deletion (nts 11,981 to 13,953), from Ψ-*ant(6′)-Ia* to *orf16*.

**Table 2.** Annotated ORFs of inserted DNA segments

ORF (aa)*	Predicted protein	Homologous protein ID/origin identity (*E* value)†	Pfam domain (aa)‡ (*E* value)
**Segment 1**			
*orf1* (267)	ATP-binding protein		AAA domain (24–168) (1.4e−14)
*orf2* (475)	DNA integrase		HTH_OrfB_IS605 (20–66) (6.1e−6) rve (155–265) (5.3e−7) Mu-transpos_C (352–410) (2.2e−13)
*orf3* (194)	DNA resolvase (invertase)		Resolvase (58–192) (9.6e−42)
*orf4* (680)	Histidinol-phosphatase, putative	MVH72510.1/*S.aureus* 572/573 (99%) (0.0)	
*orf7* (408)	Cell wall protein containing LPxTG motif, putative		Gram_pos_anchor (373–408) (8.74)
*orf8* (145)	ATP-binding protein, truncated		AAA domain (5–46) (6.6e−4)
**Segment 2**			
*orf1* (226)	IS*1216E*, transposase, IS*6* family	AAC44739.1 *E.faecium* 223/225 (99%) (3e−171)	
*orf2* (94)	Replication initiation protein, putative		
*orf3* (215)	Chloramphenicol acetyltransferase		CAT (1–205) (3.5e−68)
*orf4* (264)	Replication initiation protein		Mob_Pre (1–100) (6.3e−31) Rep_trans (119–178) (3.0e−5)
*orf5* (282)	Streptomycin adenylyltransferase Str		Adenyl_transf (1–277) (3.9e−94)
**Segment 3**			
*orf1* (244)	Class I S-Adenosyl-l-methionine (SAM)-dependent methyltransferase		Methyltransf_11 (47–141) (6.2e−23)
*orf2* (289)	Nucleotidyltransferase		NTP_transf_2 (11–111) (4.6e−8)
*orf3* (74)	Transcriptional regulator, putative		HTH_26 (7–63) (1.1e−10)
*orf4* (359)	IS*Vlu1,* transposase, IS*L3* family	QZN88035.1/*Vagococcus lutrae* 264/358 (74%) (0.0)	
*orf5* (267)	Lincosamide nucleotidyltransferase Lnu(B)		LinB-like_C (141–264) (2.2E−51)
*orf6* (494)	ABC-F type ribosomal protection protein Lsa(E)		ABC_tran (22–146) (2.9e−13) ABC_tran (326–451) (1.7e−15)
*orf7* (144)	DNA recombinase, truncated	WP198518244.1/*E.faecium* 144/144 (100%) (7e−97)	
*orf8* (152)			
*orf9* (269)	Aminoglycoside nucleotidyltransferase		Polbeta (18–100) (3.7e−08)
*orf10* (112)	Adenine phosphoribosyltransferase		Pribosyltran (24–91) (0.19)
*orf11* (237)	Aminoglycoside 6-adenylyltransferase, truncated	AAK62560.1 *E. faecium* 136/164 (83%) (3e−98)	Adenyl_transf (69–226) (1.0E−57)

*The number of amino acids of the predicted protein is shown in parentheses.

†Determined by compositional matrix adjustment.

‡Numbers in parentheses indicate the region of the protein homologous to the Pfam domain.

### Tn*7086* integration site in *E. faecalis*

**Fig. 2.**
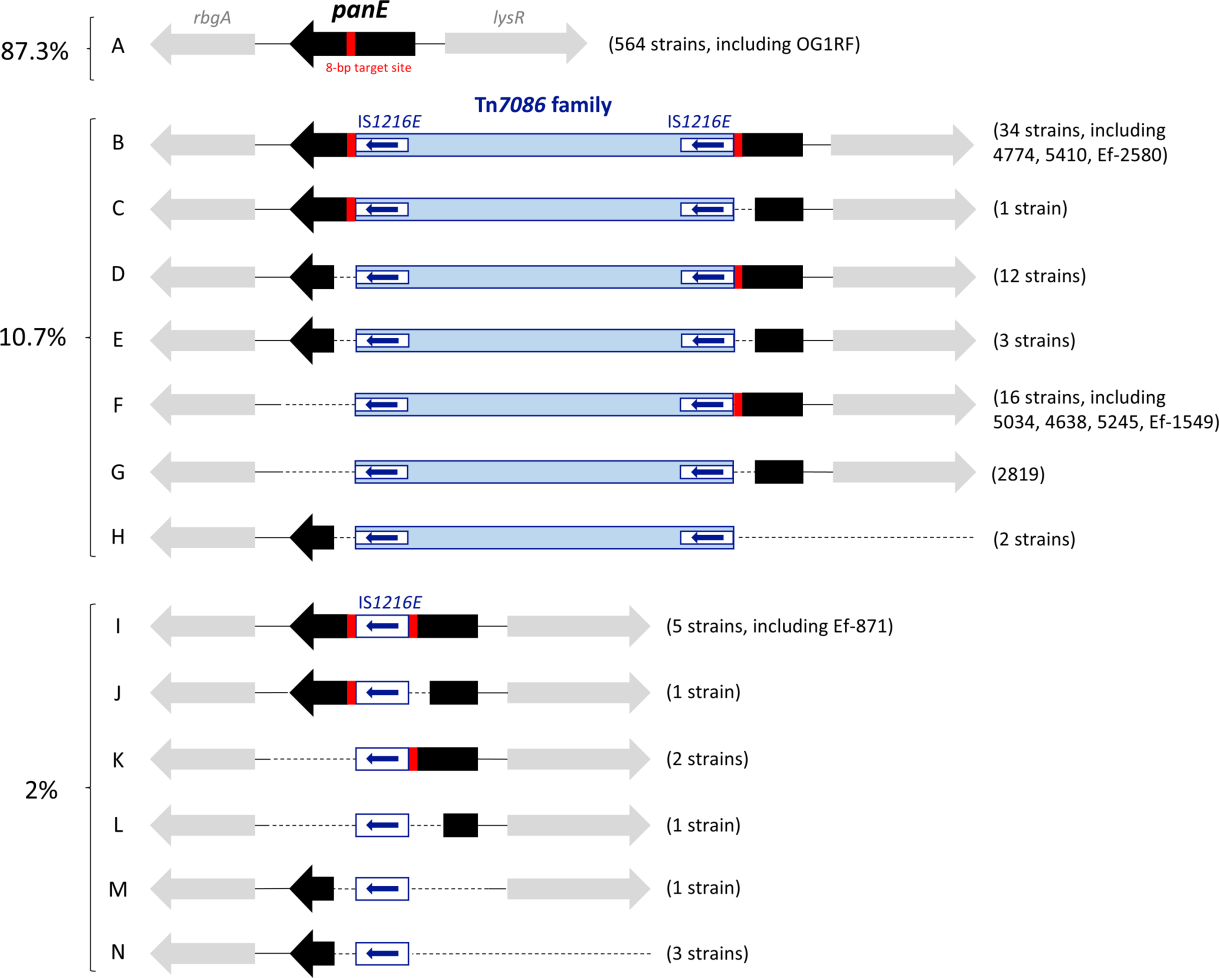
Tn*7086* integration site. Tn*7086* integrates downstream of a conserved 8-bp sequence (red box) within the 942 bp *panE* gene located between *rbgA* and *lysR* in the chromosome of *E. faecalis*. Analysis of our 9 genomes and the other 637 genomes available in public databases revealed 14 different genetic structures of the *panE* locus (structures A–N). A conserved copy of *panE* was observed in 564 isolates, including the reference strains OG1RF (structure A). The presence of Tn*7086* family members or IS*1216E* is associated with an 8-bp integration site duplication in 39 strains (structures B and I), while in 43 strains, deletions containing or not the 8-bp integration site were observed (remaining structures). Deletions involved the 5′ or 3′ ends of *panE* as well as larger regions, located upstream or downstream of the *panE* locus. Deleted regions are reported as dashed lines. The figure is not scaled.

Tn*7086* is integrated downstream of an 8-bp GC-rich specific target site (AGCCAGCG), spanning nts 509 to 516 of the *panE* gene, which is located between the *rbgA* and *lysR* genes on the Ef-1549 chromosome and codes for a 2-dehydropantoate 2-reductase involved in pantothenate biosynthesis (Fig. 2). In addition, the genome of *E. faecalis* contains one or two *panE* paralog genes located at different loci, with 70% nucleotide identity shared between each other and with the *panE* gene that serves as the integration site for Tn*7086*. A genome-wide analysis of the Tn*7086* integration site was performed on our 9 *E. faecalis* clinical isolates and the 637 complete *E. faecalis* genomes available in the NCBI database (accessed on 13 February 2025), using as a query the OG1RF 942 bp *panE* gene located between *rbgA* and *lysR*. A conserved and intact copy of the *panE* gene was found in 564 out of 646 genomes (87.3%), while in the remaining 82 genomes (12.7%), *panE* was disrupted (Fig. 2). Disruption of *panE* occurred due to the integration of a Tn*7086* transposon family member in 69 genomes (10.7%) or IS*1216E* in 13 genomes (2%) and resulted in different genetic structures. In 39 genomes, integration led to duplication of the 8 bp target site (Fig. 2, structures B and I). Alternatively, the integration led to a deletion of (i) 65 to 127 nts located upstream of the 8-bp target site in 2 genomes (structures C and J); (ii) 11 to 307 nts downstream of the target site in 12 genomes (structure D); (iii) 555 nts downstream of the target site, containing the last 426 nts of *panE* CDS in 18 genomes (structures F and K); (iv) 623 to 829 nts containing the last 494 to 700 nts of *panE* CDS including the 8-bp target site in 2 genomes (structures G and L); (v) 324 to 482 nts of *panE* CDS including the 8-bp target site in 3 genomes (structure E); and (vi) 675 to 20,366 nts containing the first 627 to 849 nts of *panE* CDS including the 8-bp target site and 48 to 19,393 nts located upstream of *panE* CDS in 6 genomes (structures H, M and N).

### Excision and circularization of Tn*7086* family transposons

PCR and sequencing analyses of genomic DNA from eight clinical isolates carrying Tn*7086* demonstrated that pseudo-compound transposons of the Tn*7086* family can excise from the bacterial chromosome (Fig. S1 Supplementary Material 1). Excision resulted in the formation of a circular translocatable unit where the left and right ends are joined by one copy of IS*1216E*. Upon excision of Tn*7086* or Tn*7086*-like elements, a single copy of IS*1216E* remained integrated into the *panE* locus. The number of circular intermediates of Tn*7086* family members varied across the clinical isolates, ranging from 1.24±0.03 to 22.4±17.7 copies per 10^6^ chromosomes in isolates Ef-1549 and 5245, respectively. The number of chromosomal sites containing a single copy of IS*1216E* varied from 6.11±2.18 to 7280±967 copies per 10^6^ chromosomes in isolates 4774 and 5245, respectively (Table 3).

**Table 3.** Real-time PCR quantification of the Tn*7086* transposon family elements, circular translocatable units and reconstituted integration sites

Strain*	Circular translocatable units†	Reconstituted integration sites†
Ef-1549	1.24×10^−6^ (± 3.71×10^−8^)	2.79×10^−5^ (± 6.25×10^−6^)
4638	1.54×10^−5^ (± 4.05×10^−6^)	6.72×10^−5^ (± 1.51×10^−5^)
2819	3.24×10^−6^ (± 1.81×10^−7^)	5.88×10^−5^ (± 3.22×10^−6^)
Ef-2580	3.09×10^−6^ (± 5.21×10^−7^)	2.65×10^−5^ (± 1.19×10^−6^)
4774	6.98×10^−6^ (± 3.62×10^−6^)	6.11×10^−6^ (± 2.18×10^−6^)
5245	2.24×10^−5^ (± 1.77×10^−5^)	7.28×10^−3^ (± 9.67×10^−4^)
5034	5.09×10^−6^ (± 6.67×10^−8^)	1.30×10^−5^ (± 4.14×10^−6^)
5410	1.30×10^−6^ (± 1.64×10^−7^)	7.23×10^−6^ (± 6.98×10^−7^)

*Ef-1549 strain carries the Tn*7086* element.

†Concentration was expressed as number of copies per chromosome. Results are reported as mean±sd from two technical replicates. Upon excision of the Tn*7086* transposon family elements, a copy of IS*1216E* remained in the integration site.

## Discussion

In this study, complete genome sequence analysis of 9 *E. faecalis* clinical strains from both localized and systemic infections allowed us to detect and characterize the novel Tn*7086* family of IS*1216E*-flanked pseudo-compound transposons, carrying the aminoglycoside resistance *aac(6′)-aph(2*″) gene. Transposons (i) have specific chromosomal integration sites located in the *panE* gene, (ii) have a mosaic structure, (iii) range in size from 15.4 kb up to 35.3 kb and (iv) contain three DNA segments, of which two carry additional antimicrobial resistance genes. The element of isolate Ef-1549 was considered the ancestral element of the Tn*7086* family, while other family members likely originate through insertions and deletions. Composite transposons carrying antibiotic resistance determinants, such as Tn*5* (kanamycin resistance) [40], Tn*9* (chloramphenicol) [41], Tn*10* (tetracycline) [42] and Tn*4001* (gentamicin) [43], are well documented in *E. faecalis*, whereas only the IS*1216E*-flanked pseudo-compound transposon Tn*7515*, carrying linezolid resistance genes and integrating into plasmid pQZ076-1, has been described [44]. *E. faecalis* genomes generally contain two or three *panE* paralog genes (<70% nucleotide identity) encoding 2-dehydropantoate 2-reductase, an enzyme crucial for pantothenate biosynthesis, which is a precursor of CoA essential for energy and lipid metabolism [45,46]. Of these paralogs, two are chromosomally encoded, while the third may be present on the chromosome or on a plasmid. Across 646 complete *E. faecalis* genomes, the *panE* paralog located between *rbgA* and *lysR* was disrupted by IS*1216E*-flanked Tn*7086* family members or IS*1216E* insertions at high frequency (82/646 genomes, 14.5%), consistent with a clonal spread of the elements. The *panE* locus exhibits extensive structural variability (14 different structures), including deletions at both the 5′ and 3′ ends, likely mediated by IS*1216E* as already described for IS*26* [47]. Since at least two copies of *panE* are present in the *E. faecalis* genome, it is likely that its function is highly constrained and is not lost upon Tn*7086* insertion. Consistently, *panE* mutants obtained in *Francisella novicida* become auxotrophic for pantothenate and can be complemented by either of the two *panE* copies of *E. faecalis* V583 [46]. DNA sequence analysis indicated that, like *E. faecalis* conjugative plasmid pRE25, Tn*7086* has a mosaic structure containing multiple antibiotic resistance determinants, including *acc(6′)-aph(2*″), *erm*(B) and *ant(6′)-Ia–sat4–aph(3′)-IIIa*, which are typically found on different transposons [34,36]. The presence of plasmid-encoded genes, such as the toxin–antitoxin system, the plasmid partition gene and *panE* itself, suggests that the Tn*7086* structure could derive from a plasmid region mobilized by the IS*1216E* elements [48]. Additionally, the long direct repeats containing *topB*, *ant(6′)-Ia* and *erm*(B) genes indicate a previous homologous recombination event, which drove the sequential acquisition of genetic material [49]. Comparison of Tn*7086* with the other seven Tn*7086*-like elements characterized in this study highlighted 5′ and 3′ end deletions mainly related to IS*Ssu5* excision and, possibly, autocatalytic RNA activity of group II intron (*orf25*), respectively [33]. Tn*7086*-like elements can host ‘passenger’ DNA, such as two segments carrying antimicrobial resistance determinants (Fig. 1b), with a DNA acquisition mechanism similar to integrons of Gram-negative bacteria [50,51]. IS-bound structures, formed by members of the IS*6* and IS*26* families such as IS*256* and IS*1216E*, mobilize through a mechanism involving the formation of cointegrates and circular translocatable units [14,52,54]. PCR analysis confirmed the formation of translocatable units of Tn*7086* carrying a single copy of IS*1216E* in *E. faecalis* isolates, whereas the other IS*1216E* copy remained in the chromosomal *panE* integration site upon excision. The ability of Tn*7086* family members to excise as translocatable units suggests potential intracellular mobilization from chromosomes to plasmids and vice versa. The simultaneous presence of conjugative plasmids in the same cell hosting Tn*7086* may favour the element’s horizontal transfer, through integration into the plasmid or by *in trans* mobilization using the plasmid conjugation machinery.

## Supplementary material

10.1099/mgen.0.001680 Supplementary Material 1.

## Abbreviations

AME: aminoglycoside-modifying enzyme
IS: insertion sequence
MGEs: mobile genetic elements
MIC: minimal inhibitory concentration
NCBI: National Center for Biotechnology Information
qPCR: quantitative PCR
