# Supplementary material for: The novel pseudo-compound transposon Tn7086 carries aminoglycoside resistance genes in Enterococcus faecalis
Source: Microb Genom. 2026 Apr 7;12(4):001680. doi: 10.1099/mgen.0.001680 (PMC13055272; doi:10.1099/mgen.0.001680)
Supplement: Supplementary Material 1. [file mgen-12-01680-s001.pdf]

Table S1. *E. faecalis* strains and their relevant properties.

Strain	Isolation site	Resistance phenotype[a]					Resistance determinant	Reference
		GEN	STR	ERY	KAN	CHL		
2819	Semen	R	S	R	R	S	*ant(6')-Ia–sat4–aph(3')-IIIa*, *erm*(B), *aac(6')–aph(2'')*	Ricci *et al.*, 2018
4638	Semen	R	S	R	R	S	*ant(6')-Ia–sat4–aph(3')-IIIa*, *erm*(B), *aac(6')–aph(2'')*	Ricci *et al.*, 2018
5245	Semen	R	S	R	R	S	*ant(6')-Ia–sat4–aph(3')-IIIa*, *erm*(B), *aac(6')–aph(2'')*	Ricci *et al.*, 2018
5410	Semen	R	S	R	R	R	*ant(6')-Ia*, *erm*(B), *aac(6')–aph(2'')*, *cat*, *str*	Ricci *et al.*, 2018
5034	Vagina	R	R	R	R	S	*ant(6')-Ia–sat4–aph(3')-IIIa*, *erm*(B), *aac(6')–aph(2'')*, *lnu*(B)–*lsa*(E), *ant(9)*	Ricci *et al.*, 2018
4774	Vagina	R	R	R	R	R	*ant(6')-Ia–sat4–aph(3')-IIIa*, *erm*(B), *aac(6')–aph(2'')*, *cat*, *str*, *lnu*(B)–*lsa*(E), *ant(9)*	Ricci *et al.*, 2018
Ef-1549	Blood	R	S	R	R	S	*ant(6')-Ia–sat4–aph(3')-IIIa*, *erm*(B), *aac(6')–aph(2'')*	This study
Ef-2580	Blood	R	S	R	S	R	*ant(6')-Ia–sat4–aph(3')-IIIa*, *erm*(B), *aac(6')–aph(2'')*, *cat*, *str*	This study
Ef-871	Blood	S	S	S	S	S	-	This study
OG1RF	Saliva	S	S	S	S	S	-	Dunny et al., 1978

[a]GEN, gentamicin; STR, streptomycin; ERY, erythromycin; KAN, kanamycin; CHL, chloramphenicol. Breakpoints were adopted from EUCAST or CLSI.

Table S2. Oligonucleotide primers.

Name	Sequence (5' to 3')	GenBank ID[a], nucleotide positions	Target / strain
IF943	ACCAAGAATATCGTCGTGGT	CP002621, 5120-5139	*gyrB* / *E. faecalis*
IF1495	GTCAGCTTCAATGGCATCAT	CP002621, 6525-6506	
IF1344	GCCTGTTCACGAGCCAATTT	CP002621, 1430445-1430464	*panE* locus / *E. faecalis*
IF1345	CGCTATGGGCAGTCGCTTT	CP002621, 1431973-1431955	
IF1404	GCCATTTTCAACCAACCTCTAA	ND, 1551950-1551971	Tn*7086* circular forms / Ef-1549
IF1405	CGACCTACTTAGACCCTCAAA	ND, 1530819-1530799	
IF1404	GCCATTTTCAACCAACCTCTAA	CP181229, 1555355-1555334	Tn*7086*-like circular forms / 4638
IF1405	CGACCTACTTAGACCCTCAAA	CP181229, 1576486-1576506	
IF1396	GGCACAATCACGGTAACTCAA	CP181238, 1560295-1560315	Tn*7086*-like circular forms / 2819
IF1418	ACACCCGAACAGTTTAAGGATA	CP181238, 1528105-1528084	
IF1406	TCCTGAAGTGATTACATCTGTA	CP181216, 1547193-1547214	Tn*7086*-like circular forms / 5245
IF1418	ACACCCGAACAGTTTAAGGATA	CP181216, 1530173-1530152	
IF1482	TTTGGAAGAAAGTATCTGCCTA	CP181214, 1556827-1556806	Tn*7086*-like circular forms / 5410
IF1400	GTGTGAGAGATAGCAATAGATTTA	CP181214, 1568794-1568817	
IF1418	ACACCCGAACAGTTTAAGGATA	CP181220, 1469546-1469525	Tn*7086*-like circular forms / 5034
IF1484	TGCTTCTAAGTCTTATTTCCATAA	CP181220, 1495850-1495873	
IF1418	ACACCCGAACAGTTTAAGGATA	CP181226, 1539909-1539888	Tn*7086*-like circular forms / 4774
IF1400	GTGTGAGAGATAGCAATAGATTTA	CP181226, 1571687-1571710	
IF1418	ACACCCGAACAGTTTAAGGATA	ND, 1543525-1543504	Tn*7086*-like circular forms / Ef-2580
IF1400	GTGTGAGAGATAGCAATAGATTTA	ND, 1564652-1564675	

[a]ND, genome sequence not deposited in GenBank.

(a)

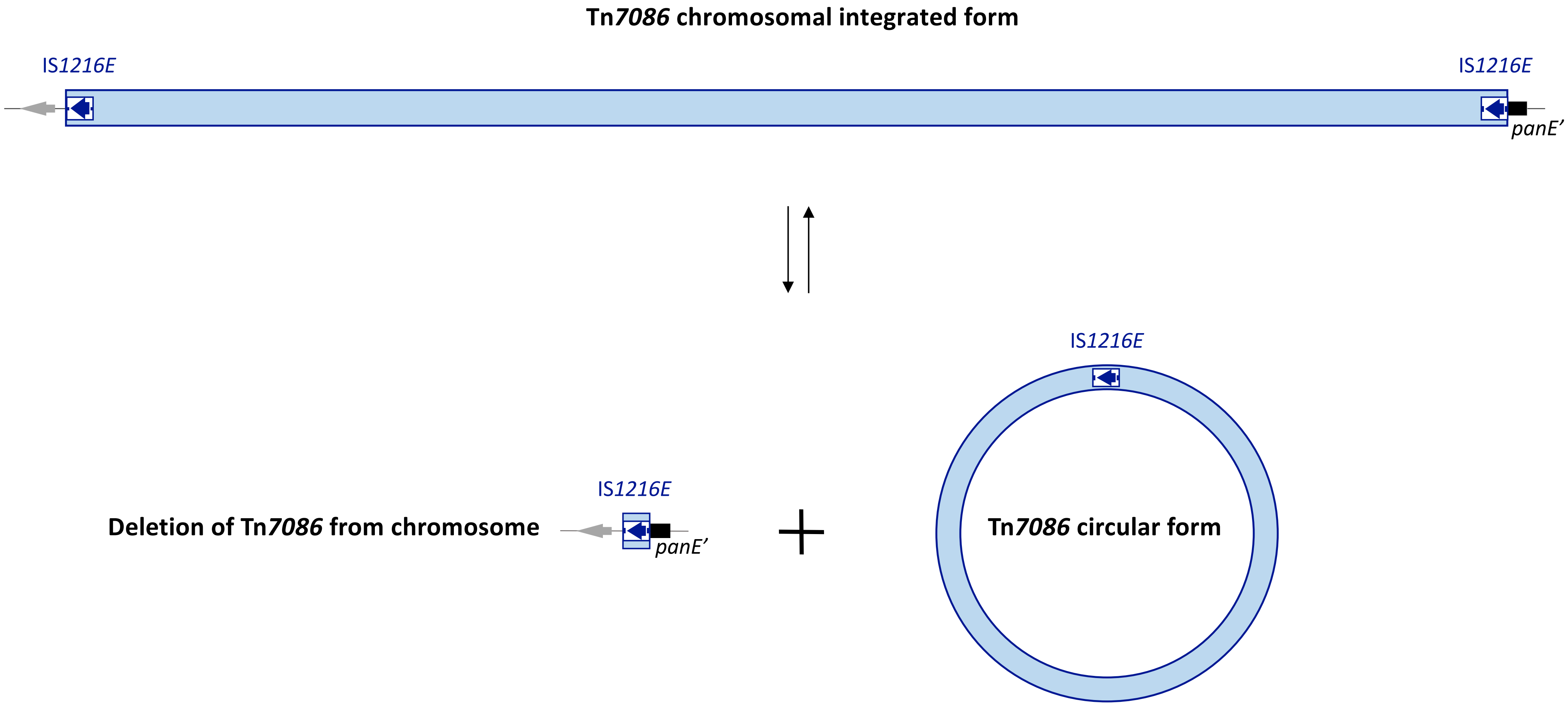

(b)

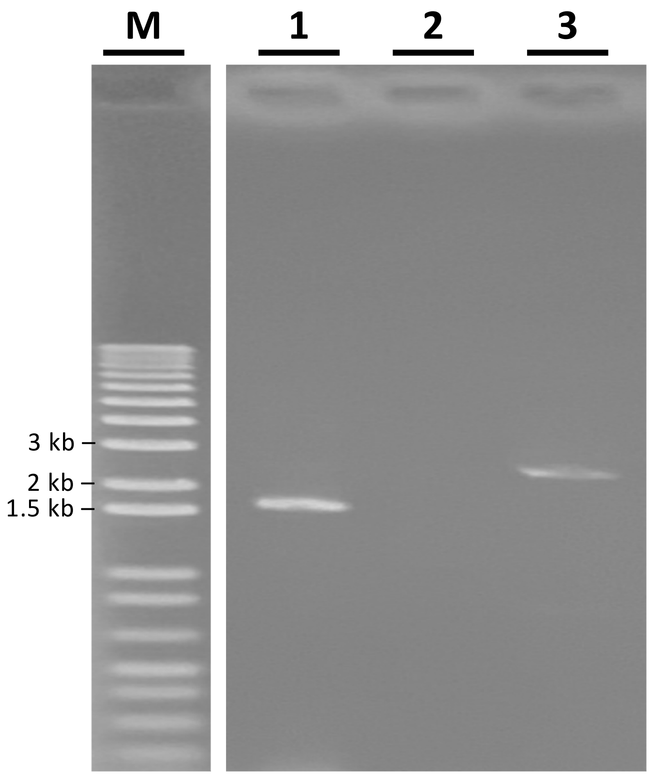

Figure S1. Schematic representation (a) and PCR detection (b) of Tn*7086* excision in *E. faecalis.* (a) The integrated form of Tn*7086* of *E. faecalis* Ef-1549 is flanked by two identical copies of IS*1216E* (boxed blue arrows). The transposon is able to excise from the bacterial chromosome leaving one copy of IS*1216E* in the chromosome and producing circular forms containing the other copy of IS*1216E*. This scheme is valid for all Tn*7086* family members. (b) Agarose gel electrophoresis of PCR products representative of a Tn*7086* element excision. Lane M, Invitrogen 1 kb Plus DNA ladder; Lane 1, amplicon of chromosomal *panE* locus containing one copy of IS*1216E* obtained with primer pair IF1344-IF1345; Lane 2, negative control; Lane 3, amplicon of the left and right ends of the circular translocatable unit obtained with primer pair IF1484-IF1418. The marker lane was repositioned for clarity and irrelevant lanes were removed.
